# Memoirs of the Life of the Late Mr. John Greenwood

**Published:** 1840-07

**Authors:** E. Bryan


### MEMOIRS

### OF THE LIFE OF THE LATE MR. JOHN GREENWOOD,

*Mechanical and Surgeon Dentist, of New-York City:—Compiled by*

E. BRYAN.—(CONTINUED.)

---

After I had been between two and three months at home, I began to get tired of a life of inactivity. There was then, in the harbor of Boston, a privateer ship, ready to start on a cruise to Barbadoes, in the West Indies, in the expectation of intercepting some of the outward-bound West India fleet of British merchantmen.

I had a great desire to go to sea, and this was too good an opportunity to be lost, so I went on board and entered as steward's mate, but I was afterwards allowed to be rated as midshipman. Our ship was called the "Cumberland," commanded by Commodore Manly. She carried eghteen guns, six pounders, and a crew of one hundred and thirty men.

We soon fell in with a prize which was a large ship or tender filled with troops from St. John, N. B., bound to Halifax, N. S ; we had no difficulty in taking her, as she had been dismasted in a gale of wind, and was then rolling about like a hogshead, and of course, unable to get away from us, or make any resistance. She had on board, clothing for the troops and some hogsheads of wine ; all the former we transferred to our vessel and some of the latter. After we had put our prize in sailing trim, by rigging her with jury masts, &c., we put a prize-master on board and ordered her to Martinique. We then pursued our course for Barbadoes, and in a few days came in sight of the island. We made the signal flag-staff late in the afternoon, and stood off from the land, with her head to the eastward, all night, under easy sail, in the expectation of seeing a prize in the morning. It was our fate, however, to be grievously disappointed. The next morning, at seven o'clock, a vessel hove in sight; she bore down towards us with all sail set; we also set all sail and stood for her, and before we could discover what she was, we found ourselves close under her larboard quarter. She proved to be the Pomona frigate of thirty-six guns, nine and twelve pounders, with three hundred men on board. We now thought it time to tack ship and make every effort to escape, but it was to no purpose,—she evidently gained upon us—and as the weather was squally, she would sometimes be near enough to give us a shot or two, which we returned from our stern chasers, aiming at her rigging, some of which we cut away, which prevented her from coming up with us, as soon as she otherwise would have done.

Here I will relate an instance of the presentiment of approaching death, which occurred in the case of the captain of the main-top, during our "running fight" with the frigate. He came down from the main-top and went into the cock-pit for a drink, which he predicted would be his last, as he said, he was certain he should never come down again alive; and so it happened. He had not returned to his post in the main-top but a few moments, before a double-headed shot from the frigate cut his body in two, which fell, a frightful spectacle, on the deck, and which made considerable impression on those who had just before laughed at what they considered his foolish prediction.

Finding that our chance of escape, became every hour less, our captain determined, as a last resort, to throw over eight of our guns; he also started some of our water, and headed our vessel three points free, which he had no sooner done, than the frigate, being right in our wake, kept her course and run up right under our larboard quarter and gave us four or five double-headed and round shot—some of which went into our rigging, and one ball striking abaft our fore chains, went through and through the vessel, which made her shake tremendously. They ordered us to strike our *rebel* colors; but our captain not much liking to give up his vessel whilst any chance, however remote, remained to be tried, conceived the idea of boarding the frigate, which he thought would come so unexpectedly upon the enemy, that we might succeed, especially as ours were all picked men, and not one on the sick list. His plan was this: Our vessel being to the leward, he meant to clap her helm down and so let the frigate run her bow right amidships of us, which would have sunk our vessel, (the sea running at the time very high,) and while our vessel was sinking, our men were to jump on board the frigate, as if to save themselves from drowning, and commence the attack, armed with cutlasses, pikes, &c. In order to accomplish this *ruse de guerre*, our captain called all hands aft, and briefly stating his plan, ordered every one to arm himself from the chests, with cutlasses, pikes, &c. But our captain had either forgotten, or had never informed himself of the extent of our arms: on opening the arm chests, there were but thirty cutlasses, and a few miserable pikes. He therefore at once ordered our colors to be struck amid the impatience of the British commander, who during this interval was threatening to fire into us and sink our vessel if we did not immediately surrender. The striking of our flag was the signal for a general plunder of the vessel by our men; they were no longer under any subordination, and every thing on board was considered fair game. The first thing they thought of was the liquor; they broke open the store room and distributed the liquor by pails full until they could drink no more; some, however, broke open the packages of the British regimental clothing, which we had taken a few days before, when a general scramble took place for these military dresses. Every one would put on his new red coat, and it was really laughable to see, in addition to their drunken "quips and cranks," the ludicrous figure they cut:—some had got coats too long—others, too short: some were too tight, while others were as loose as a sack—which, contrasting with their tarry trowsers, dirty shirts, and begrimed visages, presented such a scene as would have vanquished the gravity of an anchorite.

The British commander ordered our boat to take the captain on board the frigate, but not one of our sailors was in a condition to pay any attention to any thing but drinking and scrambling for the spoils. The petty officers at length got down the jolly boat, and in her our captain and two men were conveyed to the frigate; against her main chains, our boat was directly after, violently dashed, by the turbulence of the waves, and stove to pieces; the frigate's long boat was then sent to take the men from our vessel; as soon as they were all on board the frigate, they were mustered on the quarter deck; the master-at-arms was ordered to search every one and take away his arms and knives; all but the captain, first and second lieutenants, doctor and captain's clerk, were then ordered into the hold, where, most of them being drunk, soon fell into a sound sleep. For my part, I could not sleep for the heat, and the closeness arising from so many being stowed together in this hot climate, so, seeing the hatchway left open to let in the air, I cautiously climbed up and sat on the edge of the hatchway to enjoy the cool air, and looking round, I perceived that the sentry near me had fallen asleep, I therefore, passed by him, and went down another scuttle, into the boatswain's store room, where, treading upon something, which I at first took to be a sail rolled up, but which felt like the body of a man, either dead or asleep, I scrambled up again, faster than I went down, and resumed my former seat on the hatchway. The sentry was still asleep, and as I knew he would be severely whipped, if caught in that situation, I resolved to wake him, although I was aware that in so doing, I should forfeit my seat on the hatchway. When I awoke him, the first words he uttered were, "for God's sake go down again into the hold." I urged him to let me remain a little longer, but upon his assuring me that it was as much as his life was worth to do it, I returned to my companions.

In three days we arrived at Barbadoes; we were then landed and marched to the prison there, and as we passed along, some of the negroes threw stones at us, others called out "der goes de *new Gengelan men*, (meaning New England) dat used to bring us de fish wid only one eye" alluding to the *split mackerel*, that we had supplied them with, of which they got but *half* a mackeral for their daily allowance of fish, whereas, when they had herring served out to them they received a *whole* one, owing to the difference in the size of the two kinds of fish.

Although a little out of place, it may not be uninteresting to describe the flag of our recently surrendered vessel, the Cumberland. It consisted of the figure of a large snake, coiled into thirteen coils, emblematical of the thirteen states, each coil represented, as if *cut apart* from the others, and underneath these words, "*Join or Die*," alluding to the power, which a snake is said to possess, of preserving its life, when cut in pieces, by joining together its dissevered parts. Over the snake, was the figure of a pine tree, painted green. The flag itself was white, the snake was painted black, as was also the motto, under it. There being of course no national colors at that time, every vessel adopted such as best suited the fancy of its owner or commander.

One day the turnkey informed us that the officers of some of the men of war, then laying in the harbor, would the next day select about half of our men to be distributed on board the different vessels for service ; this news did not much please me, and five or six of us resolved in the night, if possible to break out of jail, and had nearly succeeded in cutting through the wall, when we were discovered by the patrol, and prevented from making further efforts. However, as I preferred being where I was to going on board a man of war, I determined to sham sickness, so calling to our doctor (who, together with the officers had liberty of walking in the prison yard) I told him my plan ; he gave me two emetics, one of which I took just before the arrival of the officers, but fearing it would not take effect, I also took the other, which made me so sick that I could not walk and I was left behind.

Soon after this our commodore, Manly, handed us a saw, such as is used by surgeons, with a brass back, with which we attempted to saw through a large iron-wood post but it was so thick, we could not succeed, (although we sawed on both sides,) on account of the *back*, which prevented its passing through. He told us he and the other officers intended to attempt an escape out of the jail yard into the town, and then out of the harbour by any means they could find. He said he had constructed a ladder for scaleing the walls, which he would reserve for our use, but desired that this plan should be kept secret among the petty officers, as it would be in vain for every one to attempt an escape at once.

Although we failed in our efforts, we had the satisfaction of learning, soon after, that all the superior officers made their escape out of the jail yard to the harbor, where they seized a schooner and put off to sea, passing by the men-of-war without suspicion.

In one of the apartments of the jail there were about thirty Spanish prisoners of war, who made such an incessant noise, continually quarrelling and fighting among themselves, as frequently to alarm the inhabitants of the town. One day in particular they were uncommonly turbulent and stabbed one of their companions in the breast; the jailor wished to take the ring-leader, but durst not venture alone—he therefore asked us to go in and secure him, this we readily agreed to do, and succeeded in bringing him out, and he was afterwards chained and handcuffed.

At the expiration of about five months from the time of our first arrival at Barbadoes, we were rejoiced to learn that a Cartel had arrived from Martinique, with instructions for our release. With as little delay as possible, we got on board the vessel and were soon landed at Port St. Pierre, in Martinique. Being pretty destitute of clothing and every thing else, I had made up my mind to enter on board a French privateer schooner then laying in the harbor. Fortunately however, I was recognised by an old school fellow, who hailed me on the beach and informed me that he was mate of a brig from Boston, then laying in the harbor, and that my father's cousin W——, was the commander. By his persuasion I went with him on board and saw the captain, who very kindly procured me a passage in a vessel to Piscataway, about sixty miles from Boston and gave me a hogshead of molasses to pay my expenses from thence to Boston. This vessel, being very leaky, the men had to be constantly at the pumps, at least such of them as were able, for we had the yellow fever on board, which carried off the most part of them. On our arrival at Piscataway, we found there the United States ship of war, called the Hampden, and we learned that her object was to impress men for the famous "Penobscot expedition," which was then fitting out from Boston.—Soon after our arrival, an officer of the Hampden, boarded us for the purpose of pressing our men to serve as seamen on board his vessel, but as soon as he discovered that our men were dying below of the yellow fever he left us with precipitation.

I then went on shore and sold my molasses, the proceeds of which enabled me to pay my expenses home, which being but sixty miles distant, I walked all the way, without expending much of my money, congratulating myself as I went, on the many adventures I had met, and the dangers I had escaped in the short period of my absence, it being only about six months since I had left my father's house.

My parents were delighted to see me again under their roof, and took every means to render my condition a happy one; but, not many days after my arrival, I fell dangerously sick and was confined a long time to my bed and though my recovery was deemed doubtful, I gradually got better and at length my health being thoroughly re-established, I began to long for another cruise. I entered as master-at-arms on board the ship ——, DePorter, Master, of Boston. She carried twenty eight six-pounders and one hundred and fifty men.

It was in the month of November 1778 or 9, (I have forgotten which) when we set sail with orders to cruise off the Harbor of New-York; however we soon experienced a tremendous gale which blew us into the gulf stream, and continued six days and nights incessantly so that our vessel, which had seen its best days, began to leak to such a degree that we were obliged to keep the pumps going day and night, still the leak increased, so that we had four feet water in the hold, and we expected every moment to go to the bottom. When the gale abated, the captain finding our ship was not fit for a northern latitude in the winter, concluded to sail for the West Indies. When off the Island of Jamaica we fell in with three vessels which we captured and carried into Port-au-Prince. Here we repaired our vessel as well as we could, and cruised round

Jamaica for sometime, occasionally going ashore for fresh provisions, and frequently disguising our vessel in such a manner that she could not be recognised. Sometimes we painted her sides red, yellow or black, and sometimes made signals of distress.

We captured a small Spanish schooner, she proved to be a pirate, belonging to the Island of Cuba. We took out the men and manned her ourselves. She was a convenient vessel for our purpose, mounting swivel guns. I was put second in command, on board of her. We were ordered to cruise off a small cape, near Black River, Jamaica, so that the Droghers and other small vessels would not see us until they came close upon us, when, to get clear of us they would run directly to our ship, which lay, some distance from the shore, with British colors hoisted thinking to get protection from what they supposed to be an English ship. By this device we took eleven sail of brigs and sloops, including one ship of eighteen guns, all of which we carried into Port-au-Prince.

Early one morning as we were coming out of the bite of Leogan, in the Island of St. Domingo, we observed three vessels which we took to be ships of war making towards us, but as our vessel was a very fast sailer, and had often in consequence escaped from superior force, we were not much alarmed; however we thought it most prudent to wear ship immediately and return to Port-au-Prince. We soon discovered that the three vessels were coming up with us, very fast; they brought the breeze in with them and we were soon compelled, as our only chance of escape to run our ship into a small harbor called Petit Guave, in doing which she struck on the rocks, which caused her to leak to that degree that in a short time we had four feet water in the hold.

The English ships followed us as close as they could, the fort ashore opened a fire upon them, and they left us as soon as they found out that we were on the rocks. We soon got our vessel afloat again and immediately made for Port-au-Prince, where we had been but two or three days when she filled with water, from the injury received on the rocks and sunk to the bottom and was entirely lost, beyond the chance of recovery, together with every thing on board.

By this severe disaster, I, as well as all on board lost every thing we had possessed and were left in a most destitute condition. My disposition however, was such, that I never was disheartened at any of the many misfortunes that befel me—*hope* cheered me on. I remember lately reading in Goldsmith's poems, which I borrowed of the second lieutenant, (who was somewhat of a literary man, and, I believe also something of a poet, for he was very fond of writing verses and reading poetry, during his leisure hours,)these lines on *Hope*, which I thought were *now* particularly applicable to myself:—

"Hope like the glimmering taper's light,
Adorns and cheers the way;
And still as darker grows the night,
Emits a brighter ray."

Although I had no gloomy forebodings of the future, yet I could not avoid reverting to the past, with feelings of pleasure akin to sadness.—I thought of the many pleasant hours I had passed in company with my fellow officers for they were all good and brave men and of sociable dispositions. The many "hair breadth escapes" we had had together, though they served to bind our hearts together, yet they could not prevent our being separated from one another—some going in one direction and some in another. But before we part, I will relate one of our adventures on the Island of Jamaica, which we called a *frolic* and which, although it resulted in nothing advantageous, yet it serves to show the daring spirit which animated the men of that time.

One night our first lieutenant and twelve of our officers, got into our barge, which was rowed with seven oars, and landed not far from where our ship was at anchor. We left two of our number to take care of the barge, and proceeded along shore, passing several plantations at a distance, until we had gone about three miles, when one of our party discovered a flag-staff. Our lieutenant immediately ordered us to secrete ourselves in the bushes, while he went to *reconnoitre.* This was necessary, as it was bright moonlight. In half an hour he returned, saying it was a small fort, manned with Creoles and that there was a brig laying in front of the fort, and that if agreeable to the rest he would take the fort and cut out the brig—to this we all consented and were ordered not to fire until we got close in among them, then fire and immediately draw our swords and cut them down or drive them out of the fort. We divided our force into two parties—five on one side of the road and six on the other. As soon as we arrived at the fort we rushed in; the sentry fired, but without effect, and the Creoles being surprised and panic struck, ran out at the rear entrance of the fort into a sugar cane field. In a few minutes they rallied and commenced firing their small arms at us. I proposed that we should spike the cannon of which there were seven, but we had no means of doing it, so after the lapse of about a quarter of an hour from the time of our getting possession of the fort, we concluded it best to evacuate it and retreat to our barge as quick as we could. Thereupon we all started and ran, as fast as we could, the Creoles flanking us in the bushes; we all arrived safe to our barge, but two, who gave out from fatigue (it being a three mile race) and hid themselves, in the bushes; also one of our men was wounded in the neck, as he was jumping into the barge. We rowed vigorously until we got on board our ship when we weighed anchor and stood off and soon after arrived at Port-au-Prince, which was our place of general rendezvous. One of our party who was left behind at Jamaica, soon after made his escape and rejoined us at Port-au-Prince, he informed us that there were fifty men in the fort and that when we commenced the attack, they thought from the noise we made, that we were Spaniards from Cuba, and supposed us to be at least two hundred in number, and when they found we were but eleven, they were astonished at our boldness.

